# Is Elevated Neutrophil Count and Neutrophil-to-Lymphocyte Ratio a Cause or Consequence of Schizophrenia?—A Scoping Review

**DOI:** 10.3389/fpsyt.2021.728990

**Published:** 2021-09-16

**Authors:** Asbjørn Arnesen Sandberg, Vidar M. Steen, Anja Torsvik

**Affiliations:** ^1^Norwegian Centre for Mental Disorders Research (NORMENT), Department of Clinical Science, University of Bergen, Bergen, Norway; ^2^Dr. Einar Martens Research Group for Biological Psychiatry, Department of Medical Genetics, Haukeland University Hospital, Bergen, Norway

**Keywords:** neutrophil, neutrophil-to-lymphocyte, NLR, schizophrenia, FEP, antipsychotic, psychosis

## Abstract

**Background:** Several studies have found an association between elevated neutrophil count or neutrophil-to-lymphocyte ratio (NLR) in peripheral blood from patients with schizophrenia. The etiology behind this effect is unknown, and it is unclear if changes in neutrophil count and NLR may be induced by antipsychotics or if these parameters relate to the diagnosis and symptoms of schizophrenia. The purpose of this scoping review was to map research that explores this association, and to identify gaps in the current knowledge base.

**Method:** The work was conducted in accordance with established methodological standards for scoping reviews. Studies on neutrophil count and NLR in schizophrenia were identified through search in relevant databases, and a parallel screening procedure was performed to ensure validity and reproducibility of the search. Articles that included different comparison groups, with differences in medication status (drug-naïve or drug-free vs. medicated), current disease state (relapse vs. remission), or treatment response, were included, as well as studies evaluating the association between symptomatology and neutrophil count or NLR.

**Results:** The available literature was limited with substantial differences in aims, methods, and outcomes. In total, 13 articles were included for the synthesis of this review. Some interesting trends were identified: Neutrophil count and NLR seem to be elevated in schizophrenia patients regardless of current or past use of antipsychotic therapy. Neutrophil count and NLR correlated significantly with positive symptoms of schizophrenia. Still, these findings should be interpreted with caution due to considerable methodological differences and weaknesses in the literature, particularly concerning the blood sampling procedure.

**Conclusion:** By including longitudinal studies and by comparing patient groups based on medication status, disease state and response, our study provides a basis for dissecting the associations between increased neutrophil count or NLR and a diagnosis of schizophrenia. Further research should investigate and quantify the apparent strong correlation between neutrophil count or NLR and positive symptoms in schizophrenia, to evaluate its clinical potential to guide diagnostics, treatment, or as a predictor of outcome. This review also exposes important methodological weaknesses in the literature on neutrophil count and NLR measurements. Standardization of blood sampling and processing is crucial to reduce bias, and factors that are known to influence leukocyte levels need to be accounted for.

## Introduction

The immune system and its relation to psychosis is a field with emerging interest. Epidemiological studies have shown that individuals diagnosed with schizophrenia have a higher prevalence of autoimmune diseases and infections compared to the general population (1–3). Alterations in various immune factors have been demonstrated by genetic analyses, and elevated levels of pro-inflammatory cytokines and CRP have been reported in schizophrenia vs. healthy controls (4–7). Of particular interest, recent meta-analyses have reported increased neutrophil count in patients with schizophrenia as compared to controls (8–10). Neutrophil-to-lymphocyte ratio (NLR) is another parameter that has received growing attention with respect to the inflammatory hypothesis of schizophrenia. NLR is frequently used as a marker of the balance between two immune pathways: the innate immune system through neutrophil granulocytes, and the adaptive immune system through the lymphocytes. High NLR has been found to correlate with increased levels of cytokines and CRP, and it is used increasingly in the literature as a proxy for systemic inflammation. A correlation between NLR and pro-inflammatory cytokines has been reported for ovarian cancer (11), liver cirrhosis (12), and laryngeal cancer (13). As a predictor, increased NLR is found to correlate with short- and long-term mortality in cardiac diseases and in cancer (14, 15). Also schizophrenia patients seem to have increased NLR compared to healthy controls (9, 10). However, the causality of increased neutrophil count, NLR and other immune markers is not established, and the potential clinical utility of such findings remain to be determined.

In this review, we focus on published studies that shed light on the etiology of altered neutrophil count and NLR in schizophrenia. As the observed associations between schizophrenia and neutrophils or NLR are far from fully understood, with a limited research database, we have chosen a scoping review approach to examine and map the quantity, the variety, and the characteristics of available research on this topic (16). Scoping reviews are particularly suited for issues that need investigation and understanding of larger concepts, and where the knowledge database is heterogeneous in research aim, methodology, and outcome.

Our main question is: Are changes in neutrophil numbers in schizophrenia related to the disease state or the antipsychotic medication? To touch upon this issue, we have defined several sub-questions to be addressed:

1. How does neutrophil count and NLR relate to the diagnosis of schizophrenia?
  - Is neutrophil count or NLR different in first episode psychosis (FEP) or drug-naïve or drug-free patients compared to healthy controls?
2. What is the effect of antipsychotic drug treatment on neutrophil count and NLR?
  - Is neutrophil count or NLR different between medicated and unmedicated patients?
  - Is neutrophil count or NLR changed after initiation of antipsychotic therapy?
  - Is neutrophil count or NLR different between responders and non-responders?
3. How does neutrophil count and NLR associate with symptoms and the state of the disease?
  - Is neutrophil count or NLR different between relapse and remission phase?
  - Is neutrophil count or NLR associated with disease symptoms scores?

To address these questions, we aimed at including studies that compare FEP to controls and chronic schizophrenia patients, drug-naïve to medicated schizophrenia patients, as well as patients in relapse to patients in remission. In addition, we explored available research data on the changes in neutrophil numbers and NLR and their relation to symptoms score [The Positive and Negative Syndrome Scale (PANSS) score or equivalent]. The variables to be compared were neutrophil count and NLR. The purpose was to map existing literature that can contribute to improve our understanding of the causality of the neutrophil alterations in schizophrenia, as well as to identify potential gaps in the knowledge base.

## Method

This scoping review is conducted in accordance with the Preferred Reporting Items for Systematic reviews and Meta-Analysis extension for Scoping Reviews (PRISMA-ScR) recommendations (16). PRISMA-ScR provides guidelines for a systematic, reproducible, and transparent method of conducting and reporting scoping reviews. The PRISMA-ScR-checklist is provided in Supplementary Table 1.

### Search Strategy

The search was conducted through PubMed, Web of Science and PsycInfo, on 9 September 2020 with a repeated search on 7 June 2021 to check for studies published after the first search. Adjusted to the different bibliographic styles, the following combinations of search terms were used: “Neutrophils” OR “Neutrophil-to-lymphocyte” OR “NLR” AND “Schizophrenia” OR “Psychosis.” References from selected articles were also screened, to identify additional sources.

Inclusion criteria were studies that assessed neutrophil count or NLR in schizophrenia patients or FEP patients. Articles that only assessed granulocyte count were also included, considering that neutrophils constitute the majority of the granulocytes. To be included in the scoping review, the articles should also include either longitudinal measurements, with minimum one baseline and one follow-up measurement, or relevant comparison groups. The comparison groups of interest were studies comparing different drug classes (first-generation vs. second-generation antipsychotics etc.), medication status (drug-naïve or drug-free vs. medicated), current disease state (first episode vs. relapse vs. remission), or treatment response (responders vs. non-responders). Studies that analyzed the correlation between neutrophil count or NLR and symptoms score (by PANSS or equivalent) were also included.

Exclusion criteria were: wrong population (other diagnosis than schizophrenia or FEP, *in vitro* and *in vivo* studies), wrong publication type (case reports, reviews, meta-analyses, guidelines, letters, commentaries), wrong outcome (other measures than neutrophil count and NLR), and wrong study design (cross-sectional case-control studies with no comparisons related to medication use or disease state). Lastly, studies where full-text article could not be obtained, as well as non-English articles, were excluded from this review. Date of publication was not a criterion for exclusion.

**Figure 1:**
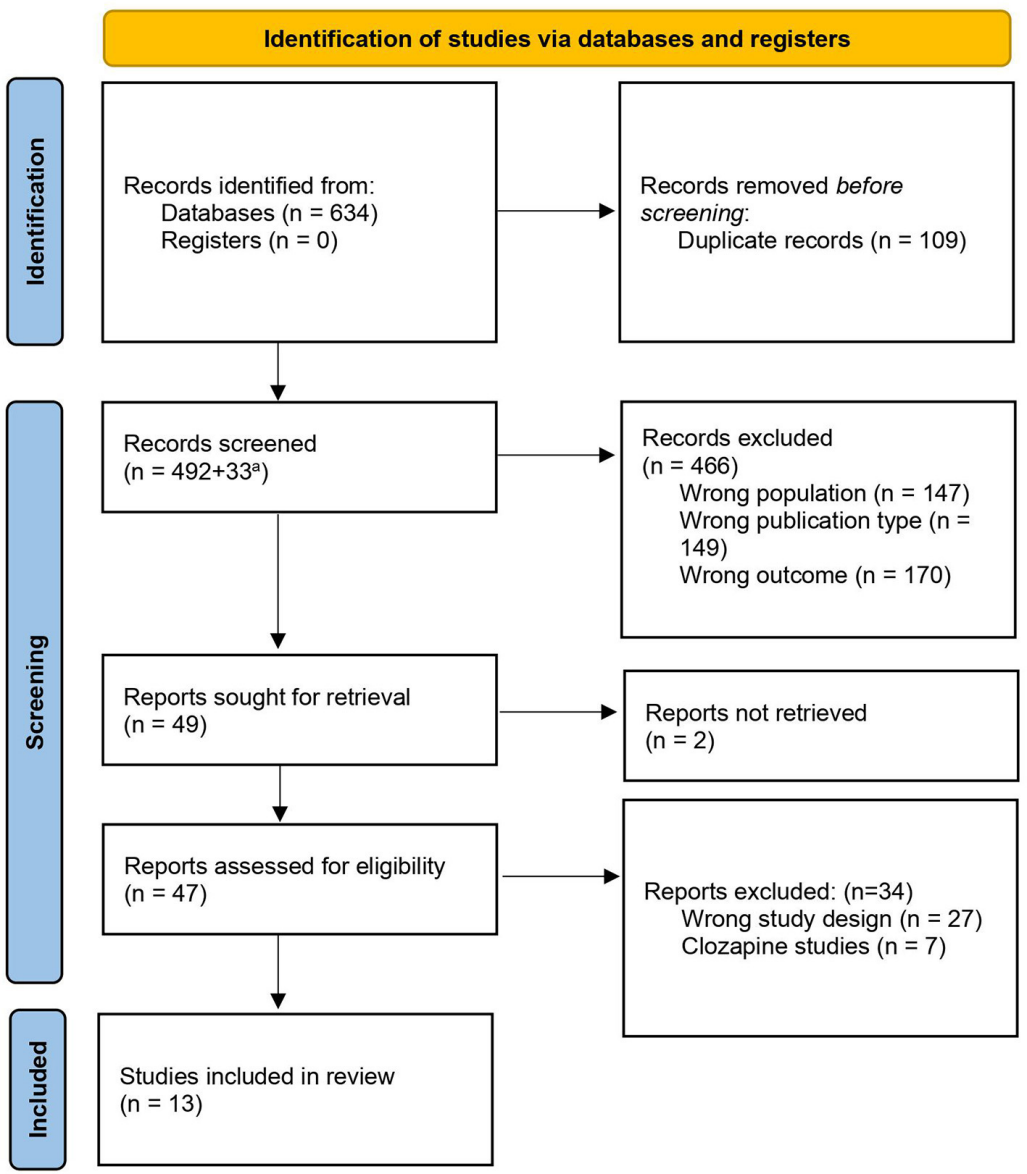
PRISMA flow diagram of the literature search and the selection process [diagram adapted from (18)]. ^a^Additional records added after repeated search on 07.06.2021.

A blinded literature screening was conducted in parallel by the first author and the senior author, to ensure reproducibility of the search. For this purpose, we used Rayyan, a web tool developed for literature screening in systematic reviews (17). Initial screening was based on title and abstract screening. Disagreement regarding inclusion/exclusion was resolved through discussion and consensus. The complete screening process is summarized in Figure 1.

We grouped the studies based on the type of information they unveiled, and their respective focus (Table 1).

**Table 1:** Included articles for this scoping review with information about study participants and comparison groups.

**References**	**Type of** **study**	**Longitudinal**	**FEP**	**Comparison** ** groups**	**Healthy** ** controls**	**NLR**	**Neutrophil** ** count**	**No. of** ** participants**
Balcioglu and Kirlioglu (19)	Retrospective cross-sectional case-control	No	No	Relapse vs. remission	Yes	Yes	No	1,063
Bustan et al. (20)	Retrospective cross-sectional case-control	Yes	No	Relapse vs. remission	No	Yes	No	366
Kovács et al. (21)	Cross-sectional cohort	No	No	Symptomatology	No	Yes	No	22
Kulaksizoglu and Kulaksizoglu (22)	Cross-sectional case-control	No	No	Symptomatology	Yes	Yes	Yes	125
Núñez et al. (23)	Multicenter cross-sectional case-control	Yes	Yes	Drug-free vs. medicated, relapse vs. remission, symptomatology	Yes	No	Yes	218
Semiz et al. (24)	Multicenter cross-sectional case-control	No	No	Drug-free vs. medicated, before vs. after antipsychotics use, symptomatology	Yes	Yes	Yes	245
Stefanović et al. (25)	Randomized trial	Yes	Yes	Drug-free vs. medicated, responders vs. non-responders, before vs. after antipsychotics use	No	No	No	78
Steiner et al. (26)	Case-control	Yes	Yes	Drug-free vs. medicated, before vs. after antipsychotics use, symptomatology	Yes	Yes	Yes	547
Zhou et al. (27)	Retrospective case-control cohort	No	No	Drug-free vs. medicated, symptomatology	No	Yes	Yes	1,144
Yüksel et al. (28)	Cross-sectional case-control	No	No	Symptomatology	Yes	Yes	Yes	105
Zorrilla et al. (29)	Case-control	Yes	No	Symptomatology	Yes	No	No	99
Arif Önder et al. (30)	Retrospective case-control	No	No	Symptomatology	Yes	Yes	Yes	64
Özdin and Böke (31)	Retrospective case-control	Yes	No	Drug-free vs. medicated, relapse vs. remission, before vs. after antipsychotics use	Yes	Yes	No	210

Although scoping reviews do not require a formal quality evaluation of included articles, we found it appropriate to evaluate certain methodological parameters. Description of study participants (Supplementary Table 1) and differences in blood sampling procedure (Table 2) were reviewed, as variation within these factors could potentially constitute a significant source of error when analyzing data on neutrophil count and NLR.

**Table 2:** Blood sampling information in the included articles.

**References**	**SAMPLING INFO**		**METHODOLOGY**		
	**Time of** **blood sampling**	**Fasting status**	**Anticoagulant**	**Time between** **sampling and analysis**	**Blood cell** **count method**
Balcioglu and Kirlioglu (19)	Within 24 h of hospitalization or on admission of outpatient unit	Not specified	Not specified	Not specified	Not specified
Bustan et al. (20)	Within 3 days of admission	Not specified	Not specified	Not specified	Not specified
Kovács et al. (21)	Not specified	Not specified	Not specified	Not specified	Not specified
Kulaksizoglu and Kulaksizoglu (22)	Not specified	12 h of fasting	EDTA	Not specified	Automated hematology analyzer (Beckman Coulter LH 780; Beckman Coulter, Brea, CA, USA)
Núñez et al. (23)	8:00–10:00 a.m.	Overnight fasting	Not specified	Not specified	Automated analyzer and if a measure was out of the normal range, cell count values were manually determined with microscopy
Semiz et al. (24)	9:00 a.m.	12 h of fasting	EDTA	Not specified	Automated blood cell counter (Abbott CELL-DYN 3700, Abbott Diagnostics Division, Abbott Laboratories, Illinois, USA)
Stefanović et al. (25)	8:00–8:30 a.m., prior to antipsychotic medication	12 h of fasting	Not specified	Not specified	Hematology analyzer ABX MICROS 60-OT (UK)
Steiner et al. (26)	8:00 a.m.	Overnight fasting	EDTA	1 h	Automated blood cell counter (XN-3000 automated counter, Sysmex Corporation)
Zhou et al. (27)	Not specified	Not specified	Not specified	Not specified	Not specified
Yüksel et al. (28)	Not specified	Not specified	Not specified	Not specified	Not specified
Zorrilla et al. (29)	At admission	Not specified	Not specified	Not specified	Automated impedance cell counter (Coulter Electronics, FL)
Arif Önder et al. (30)	Not specified	Not specified	Not specified	Not specified	Not specified
Özdin and Böke (31)	Within 2 days of admission	Not specified	Not specified	Not specified	Not specified

## Results

The literature search yielded 525 hits in total, after duplicates and non-English articles were removed. An initial parallel abstract screening resulted in 97.1% correspondence in inclusion/exclusion. We excluded 466 studies due to wrong study population (e.g., other patient groups, *in vivo* and *in vitro* studies), wrong publication type (e.g., reviews and case reports), and wrong outcome measures (Figure 1). A final screening resulted in 49 articles being proceeded to full-text eligibility assessment and further evaluation. Two could not be retrieved and 27 were discarded because of wrong study design (e.g., cross-sectional case-control studies, intervention studies, mixed diagnostic group, or wrong comparison groups). There was disagreement regarding seven articles. These were clozapine studies, and we decided to exclude them due to clozapine's potential to induce neutropenia. Thirteen articles met all inclusion criteria and were used for the synthesis of this review. An overview of the included studies is displayed in Table 1.

In summary, 12 studies were case-control studies, and one was a randomized controlled trial (RCT). Five of the 13 articles contained longitudinal measurements. Twelve of the included articles were published between 2014 and 2020, the remaining article was published in 1998. The number of participants ranged from 22 to 1,144, with a total of 3,977 study participants. Four studies had <100 participants and seven studies had more than 200 participants.

Table 2 gives an overview of the blood sampling procedures. Seven out of 13 studies clearly stated that blood sampling was done at admittance, however, the time frame differed from within 24 h to 3 days or was not specified. Only five studies confirmed the fasting status of the participants, and only four studies reported the time of day of blood sampling.

### How Does Neutrophil Count and NLR Relate to the Diagnosis of Schizophrenia?

To exclude the potential effect of medication, we first wanted to investigate if neutrophil count and NLR differ in drug-naïve or drug-free patients compared to healthy controls. We identified three studies comparing FEP-patients to healthy controls or reference values (23, 25, 26) and one that compared early-onset schizophrenia to controls (30). Núñez et al. found a significantly elevated neutrophil count in FEP patients compared to healthy controls (23). However, in this study blood sampling was not consistently drawn at admission. Some were sampled at 2 months follow-up, which implies a mix of medicated and unmedicated patients, and thus not a pure drug-naïve patient group to be compared with healthy controls.

Stefanović et al. compared granulocyte count in drug-naïve FEP-patients to a statistical reference for hematological parameters, and found that 41% of the patients had elevated granulocyte values at admission (25). Similar findings were reported by Steiner et al. (26). Blood counts at admission showed significantly higher neutrophil counts and NLR in drug-naïve FEP-patients and chronic drug-free schizophrenia patients compared to healthy controls, with 23% of the FEP and 30% of chronic schizophrenia patients having neutrophil count above the reference range, compared to 6% in the healthy controls. There was no significant difference between the various patient groups with respect to neutrophil numbers.

A study on early-onset schizophrenia also showed that patients had higher neutrophil count and NLR compared to age- and sex-matched controls (30), however, the study did not report if patients were on medication.

### Does Antipsychotic Drug Treatment Affect the Neutrophil Count and NLR?

To investigate the effect of antipsychotic medication on neutrophil count and NLR, we looked for longitudinal studies that followed patients before and after treatment, and studies that compared groups of medicated and drug-free patients. Six studies investigated the effect of antipsychotics on neutrophil numbers (Table 3). Three studies had longitudinal measurements (25, 26, 31), the others were cross-sectional (21, 24, 27). One study included a comparison based on antipsychotic response (25).

**Table 3:** Findings of neutrophil count and NLR in relation to diagnosis, antipsychotic medication, and state of the disease.

**Study focus**	**Study design**	**Neutrophil counts**	**NLR**	**References**
Relation to diagnosis	Cross-sectional	**FEP > HC**		(23)
	Cross-sectional	**FEP > HC**		(25)
	Cross-sectional	**FEP > HC**	**FEP > HC**	(26)
	Cross-sectional	**EOS > HC**	**EOS > HC**	(30)
Effect of medication	Cross-sectional	Drug-naïve FEP = drug-free SCZ	Drug-naïve FEP = drug-free SCZ	(26)
	Cross-sectional	Drug-free SCZ = medicated SCZ	Medicated SCZ = drug-free SCZ	(24)
	Cross-sectional	Drug-free SCZ = medicated SCZ	Medicated SCZ = drug-free SCZ	(31)
	Cross-sectional	**Drug-free SCZ > medicated SCZ**	**Medicated SCZ < drug-free SCZ**	(27)
	Longitudinal	**Before treatment > after treatment in FEP**		(25)
	Longitudinal	**Before treatment > after treatment in FEP**		(26)
	Longitudinal	Before treatment = after treatment in SCZ		(26)
	Cross-sectional		**Short-term > long-term antipsychothic treatment in SCZ**	(21)
	Cross-sectional	FEP responders = FEP non-responders		(25)
Association with state of disease	Longitudinal		**Relapse > remission**	(20)
	Longitudinal		**Relapse > remission**	(31)
	Cross-sectional		Relapse = remission	(19)
	Cross-sectional		**Positive correlation with PANSS-T**	(21)
	Cross-sectional	**Positive correlation with PANSS-T**	**Positive correlation with PANSS-T**	(22)
	Longitudinal	**Positive correlation with PANSS-T**		(23)
	Cross-sectional		NS correlation with BPRS	(24)
	Longitudinal	**Positive correlation with PANSS-T**		(26)
	Cross-sectional		NS correlation with PANSS-T	(28)
	Cross-sectional		**Drug-free: Positive correlation with CGI-S and BPRS-T, Medicated: Negative correlation with BPRS-N**	(27)
	Longitudinal		**Correlation between NLR at intake and reduction in SAPS**	(29)
	Cross-sectional		NS correlation with PANSS-T	(30)

*EOS, early-onset schizophrenia; FEP, first episode psychosis; HC, healthy controls; SCZ, schizophrenia; NS, not significant; PANSS-T, Positive and Negative Syndrome Scale–Total, SANS, The Scale for the Assessment of Negative Symptoms; SAPS, The Scale for the Assessment of Positive Symptoms. Significant differences are shown in bold*.

Among the cross-sectional studies, three studies found no significant difference in neutrophil count and NLR between drug-naïve or drug-free patients compared to medicated patients (24, 26, 31). However, the largest study, with 1,144 participants, found significantly higher neutrophil count and NLR in drug-free patients compared to those that had received antipsychotic therapy (27).

Different study designs were used to assess the effect of antipsychotic treatment in longitudinal studies: Steiner et al. reported a significant reduction in neutrophil count for FEP-patients (11%) and chronic schizophrenia patients (17%) after 6 weeks on antipsychotic therapy, compared to baseline measurements (26). Further analyses showed that olanzapine correlated negatively with alteration in neutrophil count in FEP-patients. Stefanović et al. divided the patients into three groups based on which antipsychotic regimen was used: first-generation antipsychotics (FGA), second-generation antipsychotics (SGA) or combined antipsychotic medication (FGA + SGA) (25). In the groups with either FGA or SGA, a significant reduction of granulocytes was found after 4 weeks of treatment. Kovács et al. compared NLR in schizophrenia patients on the basis of the length of the antipsychotic treatment (21). One group of short-term treated patients (average 3.5 ± 1.9 weeks) was compared to a group of long-term treated patients (average 8.8 ± 5.9 years). Patients on long-term antipsychotic therapy had significantly lower NLR compared to patients on short-term therapy.

Only one study grouped patients based on their response to antipsychotic treatment (25). Response was defined as at least 50% reduction of total PANSS-score. At 4 weeks follow-up, there was no difference in granulocyte count between responders and non-responders.

### How Does Neutrophil Count and NLR Associate With Symptoms and the State of the Disease?

To investigate if neutrophil count and NLR might be associated with the state of the disease, we looked at articles that compared patients in relapse vs. remission, and studies that investigated the association with symptoms score.

In total, three articles, two longitudinal and one cross-sectional study, included NLR measurements at different disease stages of schizophrenia (Table 3). The two longitudinal studies showed a significant decrease in NLR from psychosis to remission (20, 31). However, when a group of schizophrenia patients in acute exacerbation was compared with a group of patients in remission, no significant difference in NLR was found (19).

Nine studies explored the relationship between neutrophil count or NLR and psychosis symptoms score. Six studies used PANSS, two used the Brief Psychiatric Rating Scale (BPRS) (24, 27), and one used items from BPRS in combination with The Scale for the Assessment of Positive Symptoms (SAPS) and the Scale for the Assessment of Negative Symptoms (SANS) (29). Two studies also used the Clinical Global Impression-Severity scale (CGI-S).

Four studies revealed a significant association between neutrophil status and PANSS-total [Table 3; (21–23, 26)], and this was driven by a positive correlation with PANSS-P but not PANSS-N.

In a longitudinal study by Zorrilla et al. antipsychotic-naïve or antipsychotic-withdrawn schizophrenia patients were followed for 6 months (29). The authors found a correlation between relative lymphocyte and granulocyte numbers at intake to subsequent reductions in positive symptoms. More specifically, patients with a profile of lymphopenia and granulocytosis at intake had less than two-thirds reduction in SAPS-scores, while patients with normal lymphocyte and granulocyte counts at admission improved with more than two-thirds reduction in SAPS-scores.

Contrary to these findings, four studies found no correlation between neutrophil numbers and symptoms score (24, 27, 28, 30). However, Zhou et al. found that in drug-free patients, NLR was significantly associated with CGI-S and the BPRS total score, while in medicated patients NLR was negatively associated with the BPRS negative symptoms score (27).

## Discussion

To follow up on former studies that have shown increased neutrophil count and NLR in patients with schizophrenia compared to healthy controls, we made a broad systematic approach to determine if the neutrophil numbers or NLR in schizophrenia reflects the disease state or if it is a medication effect. Through different formulated sub-questions, we identified some interesting trends.

We first asked how neutrophil count and NLR relate to the diagnosis of schizophrenia. Four studies reported higher neutrophil counts in FEP or early-onset schizophrenia compared to healthy controls, indicating that this is a presenting feature of untreated disease (23, 25, 26, 30). This is in agreement with three recent meta-analyses (8–10).

We next explored the effect of antipsychotics on neutrophil count and NLR. One recent cross-sectional retrospective study reported higher neutrophil numbers in drug-free schizophrenia patients compared to medicated patients (27), while three other cross-sectional studies reported no difference between medicated, antipsychotic-withdrawn and antipsychotic-naïve schizophrenia patients (24, 26, 31). A possible explanation of this controversy is that neutrophil numbers decrease over time during antipsychotic drug exposure, as demonstrated on short-term compared to long-term antipsychotic treated patients (21) and in longitudinal studies (25, 26). This time-dependent decrease in neutrophil numbers postulates two different explanations: Neutrophil numbers may be directly decreased by antipsychotics, or indirectly by processes involved in the decrease of symptoms.

Finally, we asked how neutrophil count and NLR is associated with symptoms and the state of the disease. NLR significantly decreased when patients changed from relapse to remission (20, 31). Moreover, four of five studies found positive correlation between PANSS total score and neutrophil count or NLR. Of note, in the study that did not report correlation with PANSS (30), the patients had lower total symptoms burden than the patients in the other studies (21–23, 26). This indicates that the neutrophil numbers and NLR may be a state marker for disease severity or disease phase in schizophrenic patients, rather than an effect of antipsychotics. Interestingly, no study found correlation to PANSS negative subscale, although one study reported a negative correlation between NLR and BPRS negative symptoms score in medicated patients (27).

The observed association between neutrophils and symptoms burden is interesting. High neutrophil numbers and NLR is not a specific feature of schizophrenia but has been reported in many psychiatric diagnoses, including bipolar disorder, depression, and obsessive compulsive disorder (33–36). Stress is a trigger for mental illness and can cause increased release of neutrophils from the bone marrow by the effect of stress hormones (37, 38). How increased peripheral neutrophils relate to psychiatric disorders is less well-understood, but it may involve IL-1β-mediated neutrophil invasion and modulation of signaling in the brain [reviewed by (47)].

Since leukocyte levels fluctuate throughout the day, the time of blood sampling in essential when comparing white blood cell levels (39–41). The observed differences in leukocytes between schizophrenia patients and healthy controls in the included articles were of small magnitude, comparable to the difference seen throughout a day (42). Also the fasting status is important when comparing blood cell counts, as neutrophil count has been shown to increase after food intake while lymphocyte count decrease (32).

It is therefore a notable weakness in the literature that the description of the blood sampling procedure is often lacking in the articles, which is quite surprising when this is a major source of potential technical and biological bias. Only four studies reported the blood sampling time and only five studies informed that the study participants were fasting before blood draw. Only one study specified that antipsychotic therapy was not initiated at the time of blood sampling. In addition to this, very few studies contained information on the technical equipment or the laboratory analytical procedure of blood sampling, which allow for possible unrepresentative comparisons between and within the different studies. Neutrophils are fragile cells, and the count may be influenced by methodological variables. A recent study concluded that the neutrophil count was significantly reduced at 1 h after blood draw and when stored at room temperature (43). This study found no effect of blood collection technique and anticoagulant. However, another study showed that neutrophils were better preserved in EDTA compared to heparin and citrate (44). Automated hematology analysers and manual counts by microscopy seem to give quite comparable results, but differ in the count of immature cells (45).

The strengths and limitations of this scoping review are mainly defined by the included studies. The studies were similar with respect to inclusion/exclusion criteria for study participants. Inclusion criteria were usually schizophrenia as defined in the DSM-IV/V or ICD-10. Exclusion criteria included a list of factors or diseases that could trigger an inflammatory response, but with variation in which diseases that were included, and some were brief in the description. There were limited relevant articles available, and the studies were conducted in various ways, with differences in recruitment of study participants, duration of observation, and outcome of interest. In addition, there was large variation in the number of study participants, ranging from 22 to 1144. The number of participants and the study design are important factors when evaluating the strength of the results. Longitudinal studies are more powerful and can help to determine cause and effect relationships, while retrospective studies have more bias and need larger numbers to detect significant differences.

Another challenge when comparing different studies, concerns the patient's medication status. In the articles included in this review, different labels and definitions were used to describe the antipsychotic drug history. Several studies had no information about medication status at all. Among the patients labeled as “medicated,” only one study did comparisons based on length of treatment. None of the studies evaluated recent adherence to antipsychotic therapy. Medication compliance in schizophrenia is a constant challenge, with some studies reporting medication non-adherence to range from 20 to 89% (46). This highly variable compliance constitutes a considerable source of error when investigating antipsychotics effect on inflammatory parameters. Furthermore, the definition of being drug-free from antipsychotics varied. Two studies defined it as being off antipsychotics for more than 2 weeks, one study set the limit at 6 weeks, whereas one study did not specify any time frame. Although all antipsychotics have a half-life far below 2 weeks, it is not possible to exclude that hematological side-effects may persist even longer. Different subclasses of antipsychotics could potentially have different effects on neutrophils and lymphocytes. We also cannot rule out other confounding effects.

The scoping review methodology is also prone to its own limitations, such as the challenge to do a quality assessment. With studies of differing nature, comparisons, and weighting of results are difficult.

The trends identified in this article need to be validated. Firstly, the strength of the association between symptomatology and neutrophil count and NLR should be quantified. Furthermore, the time-course of neutrophil decrease after treatment should be further examined. Secondly, except for one study looking at granulocyte number, there are, to our knowledge, no studies comparing neutrophil count or NLR in responders vs. non-responders. It would be interesting to have studies that evaluate if this simple blood marker can be used to guide treatment of schizophrenia.

In this scoping review, we have a clear focus on methodological and technical issues that may influence the measurements of white blood cells. Further research should pursue standardized methods for blood sampling, ensuring identical timing of the procedure. The procedure should be done at the same time of day, and comparison groups should preferably be recruited in the same period/season. Factors that are known to increase leukocyte levels should be registered and accounted for, such as fasting and sleep disturbances. This would ensure more comparable and representative blood differentials and enhance our understanding of the immune system's role in schizophrenia. Further studies should also investigate whether the elevated neutrophil count and NLR is only representative for a sub-group of psychotic patients and if these patients could have beneficial effect of anti-inflammatory treatment.

## Conclusion

This scoping review investigated neutrophil count and NLR as cause or consequence of schizophrenia. We used a broad approach and included both longitudinal studies and studies with various comparison groups (medication, disease state, response, and symptoms) to identify potential causes of increased neutrophil numbers. In addition, we evaluated methodological and technical issues that may influence the measurements of blood cells.

We found that neutrophil count and NLR are increased in FEP and schizophrenia patients independent of antipsychotic use, compared to the healthy controls. A decrease in neutrophil count and NLR is reported after initiation of antipsychotic therapy, however, this may reflect an indirect effect of the parallel decrease of positive symptoms. There are substantial deficiencies in the available research, particularly concerning methodological standards and comparability of blood sampling procedures. Factors that affect the leukocyte count should be accounted for.

Further research should quantify the correlation between neutrophil count or NLR and symptomatology and investigate its clinical utility for monitoring the course of the disease.

## Data Availability Statement

The original contributions presented in the study are included in the article/Supplementary Material, further inquiries can be directed to the corresponding author/s.

## Author Contributions

AS, VS, and AT conceptualized the project. AS and AT performed the literature screening and the review process. AS wrote the first draft of the manuscript. VS and AT critically reviewed the manuscript. All authors contributed to writing and revising the manuscript.

## Funding

This work was supported by the Research Council of Norway (NORMENT CoE funding program; Grant No. 223273) and Helse Vest RHF, Norway (Grant No. F-12544).

## Conflict of Interest

The authors declare that the research was conducted in the absence of any commercial or financial relationships that could be construed as a potential conflict of interest.

## Publisher's Note

All claims expressed in this article are solely those of the authors and do not necessarily represent those of their affiliated organizations, or those of the publisher, the editors and the reviewers. Any product that may be evaluated in this article, or claim that may be made by its manufacturer, is not guaranteed or endorsed by the publisher.
